# Elastase Activity in *Aspergillus fumigatus* Can Arise by Random, Spontaneous Mutations

**DOI:** 10.4061/2010/602457

**Published:** 2010-04-11

**Authors:** Sergio Álvarez-Pérez, Jose L. Blanco, Victoria López-Rodas, Antonio Flores-Moya, Eduardo Costas, Marta E. García

**Affiliations:** ^1^Departamento Sanidad Animal, Facultad de Veterinaria, Universidad Complutense de Madrid (UCM), 28040 Madrid, Spain; ^2^Departamento Producción Animal (Genética), Facultad de Veterinaria, Universidad Complutense de Madrid (UCM), 28040 Madrid, Spain; ^3^Departamento Biología Vegetal (Botánica), Facultad de Ciencias, Universidad de Málaga, 29071 Málaga, Spain

## Abstract

*Aspergillus fumigatus* Fresenius has the capacity to degrade elastin (the principal protein of the lungs) and it is considered that elastase activity (EA) is among the most important pathogenicity factors of this mold. In particular, there is a strong correlation between EA in *A. fumigatus* and invasive aspergillosis. However, EA is not universal in this mold, and it is unknown whether the capacity to degrade elastin is the consequence of physiological mechanisms and/or genetic changes (putative adaptive mutations) induced after the exposure to this substrate or, on the contrary, it is due to random spontaneous mutations that occur under nonselective conditions. In order to discriminate between these possibilities, a Luria-Delbrück fluctuation analysis was carried out on an elastase-negative (EA^−^) *A. fumigatus* strain, using as selective factor a culture medium containing elastin as the sole source of nitrogen. Here we show that the EA^−^ → EA^+^ transformation in *A. fumigatus* appears by rare, random mutations before the exposure of the strain to selective conditions. This work represents the first experimental evidence of pathogenicity factor acquisition in mycelial fungi by preselective mutation.

## 1. Introduction

The arising of new phenotypic traits in organisms, based on selection of new genetic variants due to mutations, is a central topic in Evolutionary Biology [1]. This question is especially relevant in those cases where the phenotypic trait could be involved in pathogenic or toxic processes.

 Most species from the mycelial fungus* Aspergillus* are ubiquitous saprohytes, and a few of them are pathogenic for humans. In particular, exposure to conidia from some strains of *Aspergillus fumigatus* Fresenius may lead to severe invasive infections in immunocompromised patients [2–7]. On the other hand, *Aspergillus* species may cause allergic and toxic effects in the lung in inmunocompetent persons [8–10]. It has been considered that pathogenicity in *A. fumigatus* is the result of the activity of numerous factors, including adherence systems, toxins, and extracellular enzymes [2, 6, 11–14]. Among all the factors supposedly related to pathogenicity in this mold, elastase activity (EA) must be highlighted, not only because elastin has an essential role in the structure and physiology of the lung [15, 16], but also because this activity has also been described in other important pulmonary pathogens, such as *Pseudomonas aeruginosa* [17]. However, not all the strains of *A. fumigatus* have the capacity to degrade elastin and to use this protein as source of nitrogen. 

 At present, it is unknown whether the EA of *A. fumigatus* is due to the expression of genes already present in the population (i.e., physiological adaptation) or, on the contrary, it depends on the appearance of new gene variants by mutations (i.e., genetic adaptation, an evolutionary event). Moreover, if the EA has a mutational origin, the new gene variants might appear prior to the exposure to elastin (i.e., preselective mutations) or after contact with this substrate, during nonlethal selection (adaptive mutations). Consequently, it would be of great interest to study the nature of the appearance of EA in *A. fumigatus*, and its possible influence on the pathogenicity of the mold.

 The aim in this work was to determine which of the aforementioned mechanisms is the origin of EA in *A. fumigatus*. For this purpose, a fluctuation analysis [18] was carried out with a saprophyte, elastase-negative (EA^−^) strain of *A. fumigatus* isolated from the environment. Here we show that EA^−^ → EA^+^ transformation in *A. fumigatus* can arise as result of rare, random mutations in strains without the capacity to degrade elastin, and estimate the frequency with which the EA conferring mutation appears in *A. fumigatus* populations in the absence of elastin. This represents the first experimental evidence of pathogenicity factor acquisition in mycelial fungi by preselective mutation. The evolutionary and clinical implications of this transformation are discussed.

## 2. Materials and Methods

### 2.1. Experimental Organism, Culture Conditions, and Measurement of EA

The saprophyte strain *A. fumigatus* J4 from the culture collection of our laboratory (Facultad de Veterinaria, Universidad Complutense, Madrid) was used in all the experiments. This strain was isolated from the environment, and it is characterized by its absence of EA (EA^−^). The elastase activity index (EAI) [19] was derived by measuring the ratio between the diameters of the halo of elastin lysis (due to EA) and the mycelial growth in a plate containing the solid medium with elastin described by Kothary et al. [20].

### 2.2. Fluctuation Analysis of EA^−^ → EA^+^ Transformation

**Figure 1:**
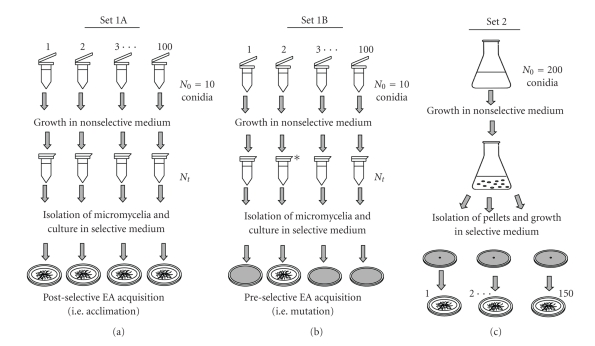
Schematic diagram of the experiment modified from the classic Luria and Delbrück [18] fluctuation analysis, and the possible results. In the set 1 experiment, different cultures (each started from a small inoculum, *N*_0_ = 10 conidia) were propagated under nonselective conditions (i.e., Czapeck-Dox broth) until micromycelia appeared, and then transferred to elastin medium. Set 1A: physiological adaptation (i.e., acclimation) or putative adaptive mutations. In this case, the number of EA^+^ nuclei in all the cultures must be similar. Set 1B: mutations occurring in the period of the propagation of cultures, that is, before exposure to the selective agent. One mutational event occurred in the propagation of culture 2, yielding EA^+^ colonies. No mutational events occurred in the other cultures, thus, no EA was observed after the transfer to the selective medium. In set 2 (control set), EA is expected in all the cultures.

Two different sets of experimental cultures were prepared, referred to as set 1 and set 2, respectively (Figure 1). Set 1 was prepared with a suspension of 10^2^ conidia mL^−1^ in PBS with 0.1% Tween 20 (PBS-T) from a culture of *A. fumigatus* strain J4 (i.e., EA^−^ strain) in Sabouraud agar (bioMérieux, Marcy l'Etoile, France). Ten conidia were inoculated into each of 100 microfuge tubes containing 900 *μ*L of Czapek-Dox broth (Pronadisa, Madrid, Spain) as nonselective medium. Tubes were then incubated at 37°C until micromycelia were obtained (23 h); this was verified microscopically. After this, the tubes were centrifuged at 16000 g for 5 minutes and the supernatants were discarded. The precipitates were washed with sterile PBS to eliminate the remaining culture broth. The tubes were centrifuged for a second time under the same conditions and the supernatants were discarded again, while the precipitates were collected by resuspension in 25 *μ*L of PBS. The contents of the 100 tubes re-suspended in PBS were inoculated into the center of 100 plates containing the solid medium with elastin.

Set 2 was prepared by inoculating 200 conidia of the same *A. fumigatus *EA^−^ strain used in set 1, suspended in PBS-T, into a 1 L Erlenmeyer flask that contained 500 mL of Czapek-Dox broth. This flask was incubated for 48 h, when macroscopic pellets appeared. After incubation, 150 pellets were picked at random with sterilized forceps and washed twice in two microfuge tubes containing 1 mL of PBS, to remove the remaining culture medium. Finally, each pellet was gently dried with a piece of sterilized filter paper and inoculated at the centre of an agar plate containing the solid elastin medium. All plates were incubated for 15 days at 37°C.

### 2.3. Confirmation of the Result from the Fluctuation Analysis

**Figure 2:**
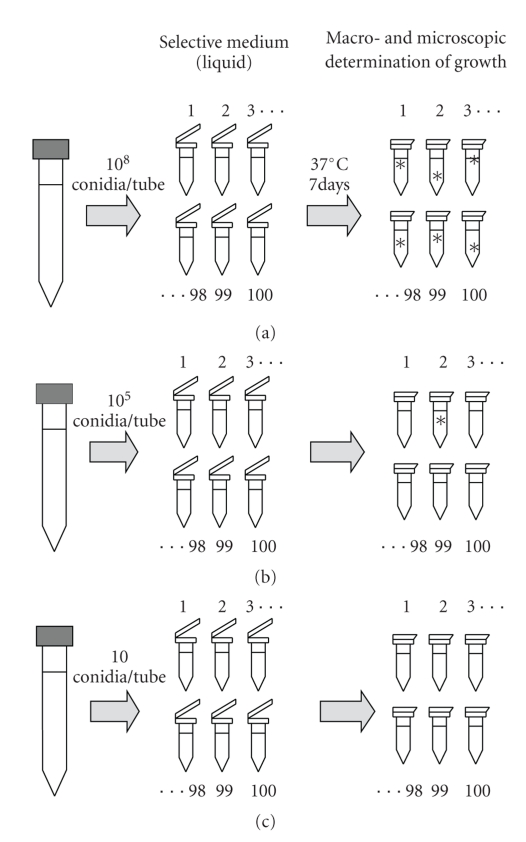
Schematic diagram of the experiment to confirm the results obtained in the fluctuation analysis.

In order to confirm the result derived from the fluctuation analysis of EA^−^ → EA^+^ transformation, an experiment relating the appearance of EA and the size of the initial inoculum was carried out (Figure 2). Due to the large number of replicas included in the experiment, microcultures in liquid selective medium were preferred instead of plate cultures (see below). The hypothesis is that if EA^−^ → EA^+^ transformation is due to physiological adaptation or putative adaptive mutations, then EA will appear in all the micromycelia growing in selective medium and, consequently, it will be independent of the inoculum size. On the contrary, EA will develop in few micromycelia if the EA^−^ → EA^+^ transformation is the result of rare, preselective mutations; consequently, the incidence of EA will be positively correlated with the inoculum size.

Three hundred microfuge tubes were prepared with 900 *μ*L of a liquid medium composed of 0.2% soluble elastin (Sigma-Aldrich, Madrid, Spain) and 0.2% yeast carbon base (Difco, Madrid, Spain) in 0.05 M borate buffer, pH 7.6. Of these 300 tubes, 100 were inoculated with 10^8^ conidia, 100 with 10^5^ conidia, and 100 with 10 conidia of *A. fumigatus* strain J4. Conidia were obtained from a suspension in PBS-T. After 7 days of incubation at 37°C, the presence of growth in the tubes was determined both macro- and microscopically.

### 2.4. Tentative Estimation of Mutation Rate

In organisms that grow as mycelia, the determination of the spontaneous mutation rate presents some difficulties, and is usually incomplete [21]. The ideal way of doing it would be to determine the mutation probability per nucleus and per generation, as done for bacteria, cyanobacteria, and microalgae. But, since in filamentous fungi the only easy way of obtaining nucleus samples is through the spores, the usual procedure is to determine the proportion of mutants among the spores formed, without considering whether the mutation occurred during spore formation or during previous nuclear divisions [21]. However, we addressed other alternative way to tentatively estimate the mutation rate of EA^−^ → EA^+^ transformation. 

 Previous to the estimation of the mutation rate, it was necessary to estimate DNA duplication rate (DDR). The DDR of *A. fumigatus* J4 was determined by DNA fluorescence using 4′, 6-diamidino-2-phenylindole (DAPI, Sigma-Aldrich). Briefly, 10 conidia from a suspension in PBS-T were inoculated in 12 microfuge tubes with 900 *μ*L Czapek-Dox; 3 other tubes containing 900 *μ*L Czapek-Dox + 8 *μ*L formaldehyde (Panreac, Barcelona, Spain) were used as controls. At the start of the experiment, 6 of the tubes inoculated with 10 conidia were fixed with 8 *μ*L formaldehyde. The other 6 tubes inoculated with conidia were growth at 37°C for 3 hours and fixed with 8 *μ*L formaldehyde. Fixed conidia or micromycelia were washed three times in PBS to remove the formaldehyde, stained with 0.15 *μ*g mL^−1^ DAPI for 1 hour and washed 12 hours in PBS. Fluorescence was measured in a spectrofluorometer (Shimazu). DDR was calculated as

(1) $$\text{DDR}\;\;={\log\;}_{e}\;\frac{\left(F_{t}/F_{0}\right)}{t},$$

where *F*_0_ and *F*_*t*_ are the fluorescence at the start and at the end of the experiment, respectively, and *t* = time (3 hours). Experiments and controls were measured blind (i.e., the person measuring the test did not know the identity of the tested sample). 

 The mutation rate (*μ*) was estimated from the proportion of cultures showing no growth in set 1 (*P_0_* estimator, the first term of the Poisson distribution), using the following expression:

(2) $$\mu \;=\;\;-{\log\;}_{e}\;\frac{P_{0}}{\left(F_{t}-F_{0}\right)},$$

where (*F*_*t*_ − *F*_0_) is the increment of fluorescence due to DNA duplication (an estimator of nuclear divisions) before inoculation in the selective medium [18, 22].

## 3. Results

**Figure 3:**
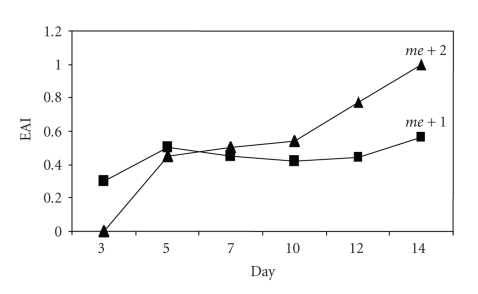
Evolution of the EAI in the EA^+^ mutants appearing in set 1 of the fluctuation analysis.

In the fluctuation analysis, after 14 days of incubation at 37°C mycelial growth was only detected in 2 of the 100 elastin medium plates inoculated in set 1, while in set 2 growth was detected in all of the 150 plates inoculated. The capacity to degrade elastin was different in the two mutants that appeared in set 1: in one of them (*me + *1) the EAI remained *ca*. 0.5 throughout the experiment, while the EAI in the other (*me + *2) increased at each measurement until the index reached a value of 1 on day 14 (Figure 3). Since their appearance in set 1, these mutants have been able to degrade elastin both in solid and liquid media, increasing their EAI in successive inoculations (data not shown). By using the expression that relates the proportion of cultures showing no growth in set 1 (*P*_0_ = 0.98) to the mutation rate (*μ*), we tentatively estimated a mutation rate of 6.17 × 10^−8^ mutants per nuclear division.

 After 7 days of incubation at 37°C, macroscopic growth was only detected in 20 of the 100 tubes inoculated with 10^8^ conidia of *A. fumigatus* J4, although microscopic mycelia were observed in 98 of them, whereas in the two remaining tubes no growth of any kind was observed. The presence of micromycelia was observed in only 4% of the tubes inoculated with 10^5^ conidia, whereas in all the tubes inoculated with 10 conidia no growth was detected.

## 4. Discussion

The occurrence of EA in *A. fumigatus* could be due to three different processes, (i) the expression of genes already present in *A. fumigatus* populations, (ii) the occurrence of adaptive mutations; or, alternatively, and (iii) the appearance of new genetic variants as result of preselective mutations. Luria-Delbrück's fluctuation analysis allows differentiation between physiological adaptation and adaptive mutations, by one hand and, on the other hand, the spontaneous arising of new genetic variants before the exposure of the saprophytic strain to a medium containing elastin as the sole source of inorganic nitrogen. Under the first possibility, if EA arises in *A. fumigatus* by physiological adaptation or adaptive mutations, the probability of the mold growing in a selective medium containing elastin as a sole nitrogen source must be similar in all the plates inoculated in set 1 and, consequently, fungal growth must appear in all of them. On the contrary, if EA arises by spontaneous mutation, each nucleus within a mycelium will have a given probability of acquiring that mutation at each division when growing in a nonselective medium. Consequently, in some cultures the mutation will take place at an early stage of the incubation under nonselective conditions, yielding a high number of EA^+^ nuclei. However, in other cultures, this mutation will occur during one of the last nuclear divisions before the transfer of the micromycelia to a medium containing elastin as unique nitrogen source, so that there will be only a small number of nuclei able to express the molecular mechanisms required to degrade elastin. Finally, the occurrence of mutations is a very rare event and, therefore, the development of the capacity to degrade elastin must have a very low probability. Thus, the number of plates in set 1 with mycelia must be very low if the EA appears as result of genetic adaptation. The strain used in the fluctuation analysis was chosen on the basis of its environmental origin and the fact that it lacks EA. The results of set 1 and set 2 of the fluctuation analysis unequivocally show that, from an environmental strain of *A. fumigatus* with an initial EAI equal to zero, EA arose in low proportion, supporting the preselective mutation hypothesis. Moreover, phenotypic differences between the mutant colonies obtained in set 1 (see Figure 3) could be evidence of a different time course, when mutations took place within the mycelia during their growth under nonselective conditions.

The results confirming the fluctuation test are also in agreement with a preselective origin of EA in *A. fumigatus*. The presence of microscopic mycelia in the majority (98%) of the tubes inoculated with 10^8^ conidia, and the absence of growth in 96% of the tubes inoculated with 10^5^ conidia, as well as in all of the tubes inoculated with 10 conidia, confirms the hypothesis of a mutational origin for the EA. If this activity were the result of a physiological adaptation process, fungal growth should have appeared in all the tubes, independent of the number of conidia inoculated. 

 If EA arises by a preselective mutation that occurs before the fungus is exposed to a medium containing elastin (such as the host's lungs), any saprophytic strain of *A. fumigatus* could acquire such ability and, therefore, become potentially pathogenic. Debeaupuis et al. [23] were unable to discriminate between clinical and environmental isolates in a study in which 879 *A. fumigatus* strains were molecularly typed. This fact has been interpreted as a proof of the hypothesis that any *A. fumigatus* strain is potentially pathogenic and could lead to a case of invasive aspergillosis if it encounters a susceptible host [5, 23]. The main practical implication of such a conclusion is that preventive measures should be applied against any environmental source of *A. fumigatus* conidia [5, 23]. Our results showing that EA is the result of preselective mutations are in agreement with this assessment. The higher the number of conidia in the environment, the greater the probability of inhaling conidia from an environmental strain that might have acquired EA by mutation sometime during its evolutionary history. Bearing in mind the ubiquity of *A. fumigatus* in the environment and its high sporulating ability [24], that possibility is more than evident. However, inhalation of conidia by immunocompetent individuals rarely has any adverse effect, since innate immune mechanisms (anatomic barriers, humoral factors, and phagocytic cells) eliminate the conidia efficiently [5, 6, 24–26].

 The mycelial growth form has been considered the main obstacle to estimating spontaneous mutation rates in filamentous fungi [21]. The usual approach is to determine the proportion of mutant conidia for a certain character that arises in a series of cultures initiated with a small inoculum [21, 27]. The main limitation of this procedure is that it cannot be distinguished whether the mutation occurred during conidia formation or during previous nuclear divisions, and therefore the analysis is incomplete unless theoretical distributions are applied, such as that proposed by Greenwood and Yule [28], which allow the estimation of mutation rates per nucleus per generation [21]. For this reason, we explored an alternative way to estimate the mutation rate of the EA^−^ → EA^+^ transformation. It should be emphasized that the mutation rate reported here was determined under laboratory conditions unlikely to be found in natural environments. Therefore, the mutational estimate might not correspond exactly to those in nature. In their natural habitats, most microorganisms, including *A. fumigatus*, likely experience fluctuations in temperature and nutrient levels. It has been demonstrated in filamentous fungi that there are inherited differences in spontaneous mutation rates according to the levels of stress in the environment, and this feature may well be adaptive. In particular, the mutation frequency of *Penicillium lanosum* and *Aspergillus niger* may be 4- and 6-fold larger in very stressful environments than in lesser stressful conditions [29]. A similar hypothesis could be assumed for *A. fumigatus* and, consequently, it could be expected that the mutation frequency is higher 10^−5^ mutation per cell cycle in the natural conditions.

 Once the EA^+^ variants appear in a population, their fate might depend on the action of different evolutive forces. For instance, in the elastin-rich medium of the host's tissues EA^+^ nuclei might be favored by positive selection and, therefore, increase their frequency. Nevetherless, even in elastin-free environments the mutation (or mutations) conferring EA might be fixed neutrally by genetic drift or other nonselective mechanisms.

 On the other hand, our results should be considered when assessing the invasiveness of different *A. fumigatus* strains. Previously we demonstrated a clear link between an EAI ≥ 1 and the ability to produce invasive aspergillosis by this mold [19]. In the present work we have found that a small percentage of the colonies within a noninvasive strain can acquire the EA by preselective mutation. Therefore, the transformation EA^−^ → EA^+^ should be taken into account when evaluating the invasiveness of each *A. fumigatus* isolate. This could be an explanation of why in our previous work we did not find a clear correlation between the EAI values and invasiveness in environmental strains, with 30.9% of strains showing EAI ≥ 1 [19].

Finally, although the molecular basis of EA is not completely understood, it seems clear that several enzymes secreted by *A. fumigatus* can degrade elastin [2, 5, 13]. Therefore, the role of spontaneous mutations in the acquisition of this trait is difficult to assess. Nevertheless, a possible explanation is that the mutation(s) conferring EA might affect a regulatory gene, probably a gene involved in nitrogen sensing or a global regulator of protease activity. Future work should address this question.

 In conclusion, this work represents the first experimental proof of pathogenic factor acquisition in mycelial fungi by preselective mutation. We believe that the study of the genetic basis of EA is of great relevance, not only for its basic interest, but also because of the possible application of the techniques presented in this article to study many others characters in mycelial fungi, specially those related to pathogenicity.
